# Preparation of Chitosan and Water-Soluble Chitosan Microspheres via Spray-Drying Method to Lower Blood Lipids in Rats Fed with High-Fat Diets

**DOI:** 10.3390/ijms14024174

**Published:** 2013-02-19

**Authors:** Yi Tao, Hong-Liang Zhang, Yin-Ming Hu, Shuo Wan, Zheng-Quan Su

**Affiliations:** 1 Key Unit of Modulating Liver to Treat Hyperlipidpemia SATCM and Lipid Metabolism Laboratory of 3rd Level SATCM, Guangdong Pharmaceutical University, Guangzhou 510006, Guangdong, China; E-Mails: taoyi8850@126.com (Y.T.); zhl198320@163.com (H.-L.Z.); huyingminggao@163.com (Y.-M.H.); wanshuoyao@hotmail.com (S.W.); 2 Department of Pharmacy, First Affiliated Hospital of Guangxi Medical University, Nanning 530021, Guangxi, China

**Keywords:** chitosan, water-soluble chitosan, microsphere, lower lipids, hyperlipidemia

## Abstract

This experiment aimed to investigate the effects of the chitosan (CTS) and water-soluble chitosan (WSC) microspheres on plasma lipids in male Sprague-Dawley rats fed with high-fat diets. CTS microspheres and WSC microspheres were prepared by the spray-drying technique. Scanning electron microscopy (SEM) micrographs showed that the microspheres were nearly spherical in shape. The mean size of CTS microspheres was 4.07 μm (varying from 1.50 to 7.21 μm) and of WSC microspheres was 2.00 μm (varying from 0.85 to 3.58 μm). The rats were classified into eight groups (*n* = 8) and were fed with high-fat diets for two weeks to establish the hyperlipidemic condition and were then treated with CTS microspheres and WSC microspheres, CTS and WSC for four weeks. The results showed that CTS and WSC microspheres reduced blood lipids and plasma viscosity and increased the serum superoxide dismutase (SOD) levels significantly. This study is the first report of the lipid-lowering effects of CTS and WSC microspheres. CTS and WSC microspheres were found to be more effective in improving hyperlipidemia in rats than common CTS and WSC.

## 1. Introduction

Chitosan (CTS), a deacetylated product of the polysaccharide chitin, is a natural biopolyaminosaccharide obtained from various organisms, including the exoskeleton of crustaceans, such as crabs, shrimps, prawns, lobsters and the cell walls of some fungi (*Aspergillus*, *Zygomicetes* and *Mucor*) [1]. The biological properties of CTS, including biocompatibility, biodegradability, low toxicity, antitumoral and antiviral activity, make it suitable for use in biomedical and pharmaceutical formulations [2]. In food and pharmaceutical industries, CTS has been proposed as a useful carrier for the delivery of essential oils and bioactive lipophilic nutraceuticals [3,4], as a dietary supplement [5], as a carrier for oral peptide and protein drug delivery [6,7] and for the controlled release of drugs [8].

In addition, CTS is a polymer containing glucosamine unit that has high positive charge densities in acidic solutions. The strong positive charge carried by the CTS makes it easy to bind negatively charged substrates, such as lipids and bile acids [9,10]. CTS also interferes with the emulsification of neutral lipids by binding them with hydrophobic bonds [11]. Previous studies revealed that the consumption of CTS had a beneficial lowering effect on plasma lipids both in animals and humans [12,13]. However, the results of some trials indicated that the effect of CTS on lower lipids was minimal and unlikely to be of clinical significance [14,15]. While CTS was believed to be of low toxicity and safe in the diet, some reports suggested that excessive intake of CTS resulted in side effects in most trials, including constipation, nausea, bloating, indigestion and abdominal pain [16]. The evidence of *in vitro* trials indicated that CTS with a smaller particle size had better cholesterol-binding capacities [17]. The pharmaceutical industry always attempts to design drugs as microspheres to control the release and elevate the bioavailability [18]. Recently, many kinds of microspheres have been prepared by the spray-drying method for narrow distribution and high yield [19]. Therefore, CTS microspheres have become one possible solution to the problem.

In this study, CTS microspheres were prepared by the spray-drying method as an alternative of CTS due to its grand bioavailability, control release and minimal side effects. In previous studies, the water-soluble chitosan (WSC) was more effective at lipid-lowering [20]. WSC is a derivative of chitosan and exhibits good solubility in water. Accordingly, it was important to focus the study on the hyperlipidemic effects of both CTS and WSC and the effect of CTS and WSC microspheres on plasma lipids in rats fed with high-fat diets.

## 2. Results and Discussion

### 2.1. Morphology of Microspheres

**Figure 1:**
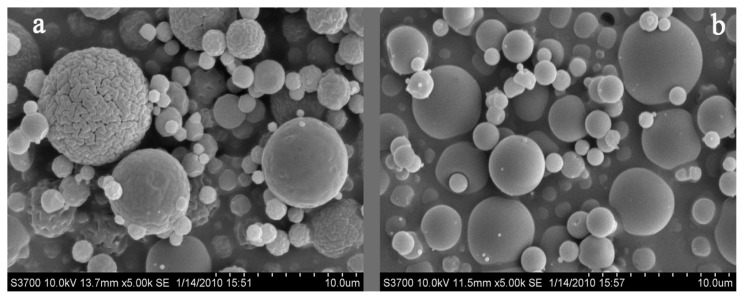
Scanning electron microscopy (SEM) microphotographs of (**a**) chitosan (CTS) microspheres and (**b**) water-soluble chitosan (WSC) microspheres obtained by spray drying (magnification 5,000×).

**Figure 2:**
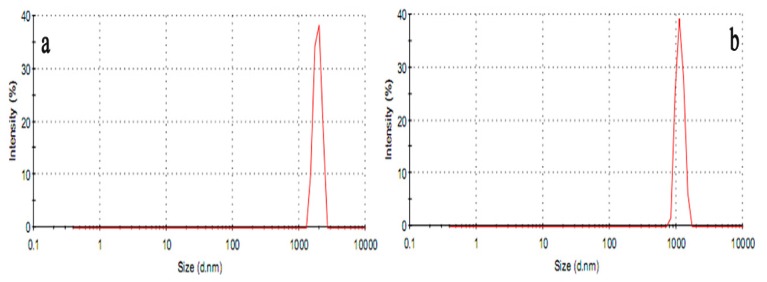
Particle size distribution (PSD) of (**a**) CTS microspheres and (**b**) WSC microspheres.

The scanning electron microscopy (SEM) micrographs of CTS and WSC microspheres are shown in Figure 1. All microspheres were found to be nearly spherical in shape, and the external surfaces of the WSC microspheres appeared smooth. The particle size distribution (PSD) of CTS and WSC microspheres is shown in Figure 2. The mean particle size of CTS and WSC microspheres was 4.07 μm and 2.00 μm and varied from 1.50 to 7.21 μm and from 0.85 to 3.58 μm, respectively.

### 2.2. Effects on Body Weight

**Figure 3:**
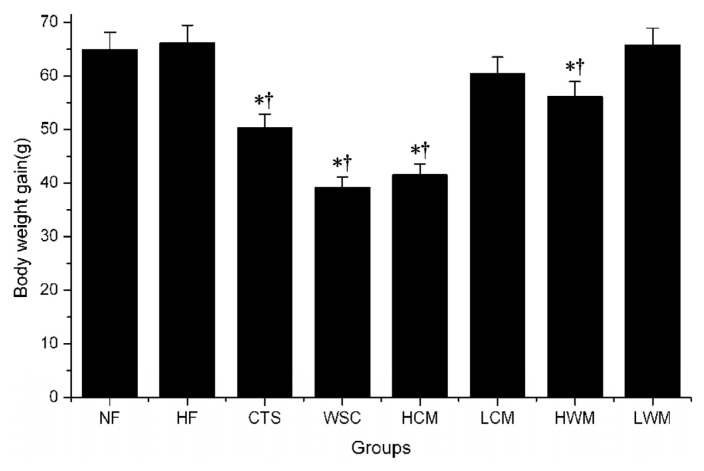
Effects of CTS and WSC microspheres on weight gain in rats fed with high-fat diets. Results are expressed as the mean ± SE of eight rats. ^*^p < 0.05, significantly different when compared with rats fed with a normal diet; ^†^*p* < 0.05, significantly different when compared with rats fed with a high-fat diets.

A significantly lower weight gain was observed by feeding the WSC and high dose CTS microspheres compared with feeding the placebos (distilled water) and high-fat emulsions (*p* < 0.05) (Figure 3). However, no significant differences in average weight gain were observed among the WSC, CTS and high dose of chitosan microsphere fed rat (HCM) groups. Although, weight gain tended to decrease in the CTS, low dose of chitosan microsphere fed rat (LCM) and high dose of water-soluble chitosan microsphere fed rat (HWM) groups, the differences were not statistically significant compared with normal diet fed rat (NF) and high-fat emulsions fed rat (HF) groups.

During the four-week experiment, daily administration of WSC (225 mg/kg/day) and high-dose CTS microspheres (450 mg/kg/day) significantly reduced weight gain. The average weight gain decreased in the other treatment groups also, but it was insignificant compared with the NF group. This indicated that WSC may prevent high-fat diet induced increase of body weight by affecting food absorption, and the CTS microspheres prepared with CTS are more effective in decreasing the weight gain than CTS. However, WSC microspheres were not shown to be more effective in decreasing weight gain compared to the WSC. This indicated that the anti-obesity mechanism of WSC differed with that of CTS for different compositions. Thus, WSC and CTS microspheres may reveal an anti-obesity action, but further study is needed to clarify this action.

### 2.3. Effects on Serum and Liver TC, TG, HDL-C, LDL-C and SOD

**Figure 4:**
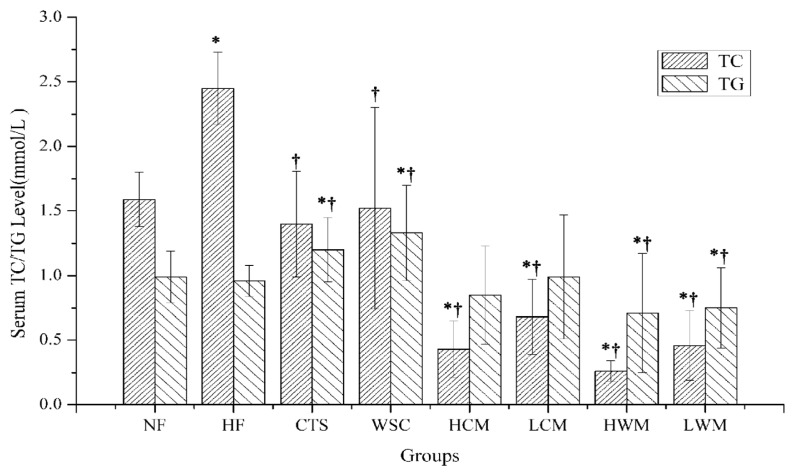
Effects on serum total cholesterol (TC) and triglycerides (TG) in rats fed with high-fat diets. Results are expressed as the mean ± SE of eight rats. ^*^p < 0.05, significantly different when compared with rats fed with a normal diet; ^†^*p* < 0.05, significantly different when compared with rats fed with a high-fat diet.

**Figure 5:**
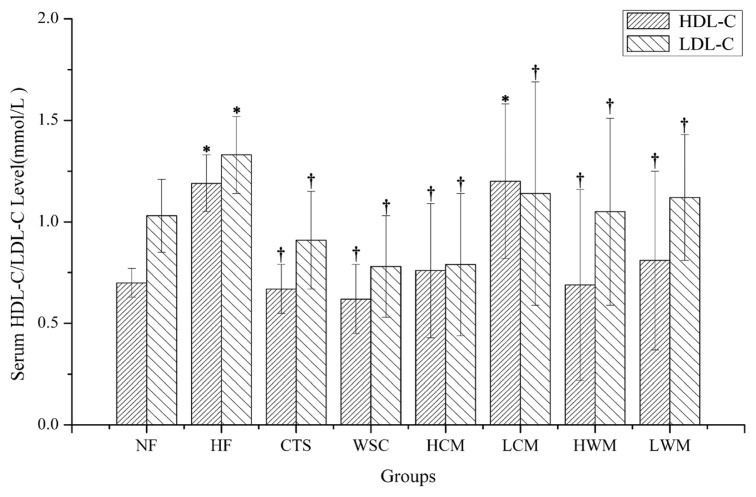
Effects on serum high-density lipoprotein cholesterol (HDL-C) and low-density lipoprotein cholesterol (LDL-C) in rats fed with high-fat diets. Results are expressed as the mean ± SE of eight rats. ^*^p < 0.05, significantly different when compared with rats fed with a normal diet; ^†^*p* < 0.05, significantly different when compared with rats fed with a high-fat diet.

The results of serum total cholesterol (TC), triglycerides (TG), high-density lipoprotein cholesterol (HDL-C) and low-density lipoprotein cholesterol (LDL-C) are presented in Figures 4 and 5. It was found that feeding of high-fat emulsions resulted in the elevations of serum TC, TG and LDL-C significantly compared with the NF group (*p* < 0.05), suggesting that hyperlipidemic condition was produced in the experimental HF group successfully. In the HCM, LCM, HWM and low dose of water-soluble chitosan microsphere fed rat (LWM) groups, serum TC was significantly reduced by feeding microspheres compared with NF and HF groups (*p* < 0.05). However, in the CTS and WSC groups, serum TC levels were only significantly reduced compared with the HF group (*p* < 0.05). The serum TG levels of rats in the HF group were significantly higher than those of the CTS and WSC microspheres-treated groups (*p* < 0.05). In contrast, the CTS and WSC groups were not significantly different compared with the HF group. When compared with the HF groups, a significant decrease of serum LDL-C was seen in the six treatment groups (*p* < 0.05). However, there was no difference in the serum HDL-C among all groups.

Generally, as the weight is fixed, the smaller the particle size, the bigger is the total surface area. CTS and WSC microspheres possess very finer particle size, which may facilitate adsorption of lipids and bile acids. In clinical experiments, either a high-dose or low-dose of CTS and WSC microspheres can decrease the serum TC, TG or LDL-C levels significantly compared with the HF group, whereas CTS and WSC can only decrease the TC significantly compared with the HF group. This is consistent with the report that CTS with a finer particle size could effectively lower the plasma lipid level in rats fed with a high cholesterol diet [21]. In the present experiment, serum TG levels were not significantly different in the CTS and WSC groups compared with the HF group. Hossain [22] noted that the reason might be related to the fact that TG was an electrically neutral lipid molecule and the positive charge of CTS could not interact with the neutral TG molecule of the diet and bile acid in the intestine. Serum HDL-C levels increased only slightly in the LCM group, and the serum LDL-C levels decreased significantly by feeding the CTS, WSC and microspheres compared with the HF group. The low levels of HDL-C and the high levels of LDL-C indicated an imbalance between cholesterol transport from the liver to the extrahepatic tissues and back to the liver [22]. Therefore, we proposed that the CTS and WSC microspheres could decrease serum TC, TG and LDL-C in hyperlipidemic patients and serve as a useful dietary supplement for preventing hyperlipidemia.

The serum superoxide dismutase (SOD) increased significantly only in the HCM and HWM groups compared with the NF and HF group (*p* < 0.05) and only slightly increased in all other groups, except the CTS group (Table 1). Serum SOD also increased by feeding high-dose microspheres compared with feeding CTS and WSC. However, there was no significant difference in the LCM and LWM groups.

There was no significant difference in liver TC, TG, HDL-C and LDL-C levels among all groups at the end of the experimental period. Liver SOD level increased significantly by feeding the high dose of CTS and WSC microspheres (*p* < 0.05). Liver SOD showed an increasing trend in the low-dose microspheres-treated groups; however, this increase was not significant compared with the CTS and WSC groups (Table 1).

SOD is one of the major free radical-scavenging systems that might play a role in removing superoxide radicals. Excessive superoxide radicals may induce lots of senile diseases, such as atherosclerosis. Any elevation in the SOD level is accompanied by a decrease in superoxide radicals [23,24]. In this experiment, the serum SOD elevated only significantly by feeding high-dose CTS and WSC microspheres compared with the NF and HF groups, and CTS and WSC could not increase the serum SOD any further. In the six treatment groups, liver SOD levels showed a significant increasing trend. When compared to the CTS and WSC groups, significant differences were seen for the HCM and HWM groups. This may be because microspheres can penetrate deeply into tissues through fine capillaries. Therefore, we could conclude that the CTS and WSC microspheres increased serum and liver SOD levels more effectively than CTS and WSC.

### 2.4. Effects on Plasma Viscosity

**Figure 6:**
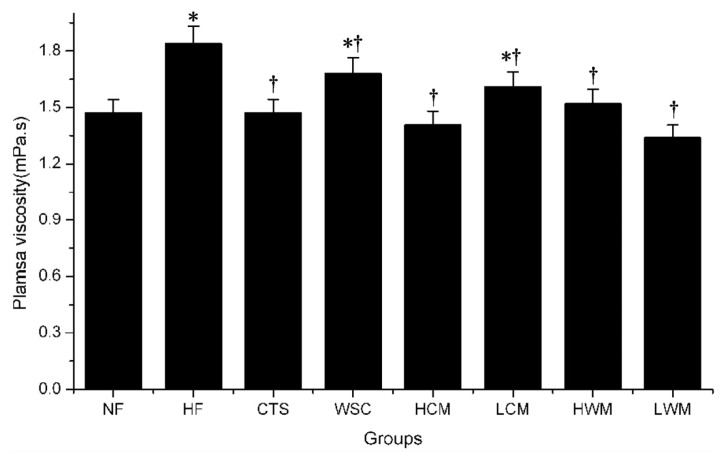
Effects of CTS and WSC microspheres on plasma viscosity in rats fed with high-fat diets. Results are expressed as the mean ± SE of eight rats. ^*^p < 0.05, significantly different when compared with rats fed with a normal diet; ^†^*p* < 0.05, significantly different when compared with rats fed with a high-fat diet.

In this experiment, the average plasma viscosity of rats in the HF group increased significantly compared with the average level for rats in the NF group (*p* < 0.05) (Figure 6). In the six treatment groups, plasma viscosity showed a significant decrease compared with the HF group (*p* < 0.05).

Plasma viscosity played an important role in the perfusion of the microvasculature and was a major determinant of endothelial shear stress [25]. It was used as a marker for different diseases in humans, such as coronary artery disease and atherosclerosis [26]. The rats’ plasma viscosity increased significantly by feeding the high-fat emulsions, and CTS and WSC microspheres reduced this increase effectively. Furthermore, the microspheres were more effective than CTS and WSC. There was no significant difference in plasma viscosity among the microsphere-treated groups, which suggested that the rats’ high plasma viscosity model used previously did not adequately simulate the human hypocholesterolemia. However, further human studies are needed to confirm this.

## 3. Experimental Section

### 3.1. Chemicals

CTS and WSC with an average molecular weight of 350 kD and 200 kD were purchased from Shandong Aokang Biotech Ltd. (Shandong, China). The viscosity was more than 200 cps, and deacetylation values were 96.2% and 85%, respectively. Total cholesterol (TC), triacylglycerol (TG), high-density lipoprotein cholesterol (HDL-C) and low-density lipoprotein cholesterol (LDL-C) kits were obtained from BioSino Bio-technology and Science Inc. (Beijing, China). Superoxide dismutase (SOD) kits were purchased from Nanjing Jiancheng Bioengineering Institute (Wenzhou, China). Unless otherwise stated, all laboratory reagents were of analytical grade.

### 3.2. Animals and High-Fat Emulsions

Male Sprague-Dawley rats weighing 200 ± 20 g were purchased from Guangzhou University of Chinese Medicine Laboratory Animal Center (Guangzhou, China). All animal protocols were approved by the Institutional Animal Care and Use Committee of Guangdong Pharmaceutical University (Guangzhou, China). They were housed in an isolator caging system in an air-conditioned animal room at 23 ± 1 °C and had free access to food and water. The high-fat emulsions were prepared by the method of our previous study [27].

### 3.3. Preparation and Characterization of CTS and WSC Microspheres

CTS and WSC microspheres were prepared by the spray-drying technique. Dissolving CTS in acetic acid (1.0% *v*/*v*) produced the solution containing 2.5% (*w*/*v*) CTS, and the WSC solution was prepared by dissolving WSC in deionized water containing 2.0% (*w*/*v*) WSC. The solutions were then spray dried using the Lab Spray Dryer L-117 (Laiheng Scientific Co. Ltd., Beijing, China) with a standard nozzle (0.7 mm). The atomizing air flow rate was 10–15 L/min, and the flow rate was 600 mL/h. The inlet temperature was controlled at 160 °C. The outlet temperature varied between 80 and 85 °C and was determined by the inlet temperature and other relative factors, such as air and liquid feed flow rates. The morphology of the microparticles was examined under scanning electron microscopy (SEM) using a Hitachi S3700N (Hitachi Ltd., Tokyo, Japan) microscope at 10 kV. The particle size and size distributions of the CTS microspheres and WSC microspheres were determined with a particle sizer (Zetasizer 3000HS Malvern Instruments Ltd., Malven, UK).

### 3.4. Experimental Procedure

The rats were fed *ad libitum* with a commercial diet for five days and were then classified into eight groups (*n* = 8): normal fat control group (NF), high fat control group (HF), chitosan control group (CTS), water-soluble chitosan control group (WSC), chitosan microsphere group (high-dose (HCM) and low-dose (LCM)) and water-soluble chitosan microsphere group (high-dose (HWM) and low-dose (LWM)).

The NF group received an equivalent amount of distilled water; the HF group received high-fat emulsions daily by oral intubation until the study ended. The other groups were administered high-fat emulsions by oral intubation for two weeks to establish the hyperlipidemic condition, and then, the CTS and WSC samples (450 mg/kg/day) were administered orally once per day to the CTS and WSC group for four weeks. Similarly, two doses (high dose 450 mg/kg/day; low dose 225 mg/kg/day) of CTS and WSC microspheres were administered orally once per day to the HCM, LCM, HWM and LWM groups for four weeks after two weeks to establish the hyperlipidemic condition. CTS, WSC, CTS microspheres and WSC microspheres (5.0 g) were dissolved with distilled water (100 mL). All groups were fed the corresponding diets in which the composition conformed to GB14924.3 (Guangdong Laboratory Animal Center, Guangzhou, China) as the basal diets during the whole experiment. Each rat was weighed once a week.

At the end of the experimental period, blood samples were withdrawn from the orbital venous plexus using a capillary tube under ether anesthesia after an overnight fast. Then, the rats were decapitated and their livers were quickly removed and weighed. The liver pieces was immediately stored at −80 °C until analysis.

### 3.5. Serum and Liver Lipids and SOD

Blood was clotted at room temperature and centrifuged at 3,000 rpm for 15 min. Serum was separated and TC, TG, HDL-C and LDL-C levels were measured with commercial assay kits using the Automated Biochemistry Analyzer AMS-18 (Beijing Option Science and Technology Development Co. Ltd., Beijing, China).

The liver TG, TC, HDL-C and LDL-C contents were measured as follows: a piece (0.1 g) of liver tissue was homogenized with chloroform-methanol (2:1, *v*/*v*, 2 mL), and the homogenate was extracted with chloroform-methanol (2:1, *v*/*v*, 3 mL) by shaking the tubes horizontally for 24 h in a shaker. The mixtures were centrifuged at 3,000 rpm for 5 min, and the upper aqueous phase was removed by suction. The liver TG, TC, HDL-C and LDL-C contents were analyzed with commercial assay kits.

The serum and liver SOD contents were analyzed with the commercially available analytical kit by the SPECORD S600 UV-Vis Spectrophotometer (Analytic Jena AG, Jena, Germany).

### 3.6. Plasma Viscosity

In order to obtain plasma, blood samples were taken from the ocular vein using a heparinized capillary tube and centrifuged at 3,000 rpm for 5 min in the Eppendorf Centrifuge 5810R (Eppendorf Co., Hamburg, Germany). The plasma viscosity was measured using the Automatic Blood Rheometer LBY-N6B (Beijing Precil Instrument Co. Ltd., Beijng, China).

### 3.7. Statistical Analysis

All data were expressed as the mean ± SE. Differences between the groups were determined by one-way analysis of variance (ANOVA) using a statistical analysis software program, SPSS (version Rel., 16.0, Spss Inc., Chicago, IL, USA). The Student-Newman-Kuels Multiple Range Test comparisons at *p* value of <0.05 were made to determine significant differences among means.

## 4. Conclusions

To conclude, the results indicated that the microspheres prepared from common CTS and WSC are effective in lowering serum lipid levels and plasma viscosity and increasing SOD levels in rats fed with high-fat emulsions. Thus, the particle sizes of CTS and WSC affect their hypocholesterolemic activities. Further clinical studies are needed to clarify the preventive effect on diet-induced obesity of the microspheres. In our previous article, the median lethal dose (LD_50_) values of CTS, WSC and CTS microspheres were >10,000 mg/kg, and the median lethal dose values of WSC microspheres were 4,080 mg/kg [28]. To date, all rats appear healthy and remain active after oral administration of the CTS, WSC and their microspheres. Therefore, CTS, WSC, CTS and WSC microspheres can be considered as safe functional polymers.

## Figures and Tables

**Table 1:** Effects on serum and liver superoxide dismutase (SOD) in rats fed high-fat diets.

Group	Serum SOD (U/mL)	Liver SOD (U/mL)
NF	83.43 ± 9.78	0.97 ± 0.34
HF	87.18 ± 14.01	0.92 ± 0.57
CTS	72.26 ± 6.10 *,†	1.65 ± 0.48 *,†
WSC	92.35 ± 6.32 *	1.26 ± 0.78 *,†
HCM	113.37 ± 11.62 *,†	4.07 ± 0.73 *,†
LCM	90.77 ± 8.49 *	2.94 ± 0.21 *,†
HWM	99.15 ± 9.66 *,†	4.98 ± 0.70 *,†
LWM	92.76 ± 10.83 *	1.73 ± 0.41 *,†

Values are expressed as means ± SE (*n* = 8).

* *p* < 0.05, significantly different when compared with rats fed with a normal diet;

† *p* < 0.05, significantly different when compared with rats fed with ahigh-fat diet.
